# Low carbohydrate diet in type 1 diabetes, long-term improvement and adherence: A clinical audit

**DOI:** 10.1186/1758-5996-4-23

**Published:** 2012-05-31

**Authors:** Jørgen Vesti Nielsen, Caroline Gando, Eva Joensson, Carina Paulsson

**Affiliations:** 1 Formerly Department of Medicine, Blekingesjukhuset, Karlshamn, 37480, Karlshamn, Sweden; 2 Department of Medicine, Blekingesjukhuset, Karlshamn, 37480, Karlshamn, Sweden

**Keywords:** low-carbohydrate, diet, type 1 diabetes, adherence, HbA1c, IFCC

## Abstract

**Background:**

Reduction of dietary carbohydrates and corresponding insulin doses stabilizes and lowers mean blood glucose in individuals with type 1 diabetes within days. The long-term adherence for persons who have learned this technique is unknown. To assess adherence over 4 years in such a group the present audit was done retrospectively by record analysis for individuals who have attended an educational course. Adherence was assessed from HbA1c changes and individuals’ own reports.

**Findings:**

Altogether 48 persons with diabetes duration of 24 ± 12 years and HbA1c > = 6.1% (Mono-S; DCCT = 7.1%) attended the course. Mean HbA1c for all attendees was at start, at 3 months and 4 years 7.6% ± 1.0%, 6.3 ± 0.7%, 6.9 ± 1.0% respectively. The number of non-adherent persons was 25 (52%). HbA1c in this group was at start, at 3 months and 4 years: 7.5 ±1.1%, 6.5 ± 0.8%, 7.4 ± 0.9%. In the group of 23 (48%) adherent persons mean HbA1c was at start, at 3 months and 4 years 7.7 ± 1.0%, 6.4 ± 0.9%, 6.4 ± 0.8%.

**Conclusion:**

Attending an educational course on dietary carbohydrate reduction and corresponding insulin reduction in type 1 diabetes gave lasting improvement. About half of the individuals adhered to the program after 4 years. The method may be useful in informed and motivated persons with type 1 diabetes. The number needed to treat to have lasting effect in 1 was 2.

## Background

Considering the detrimental effect of hyperglycemia upon all organ systems the achieving of euglycemia or near-euglycemia is essential in the care of type 1 diabetes [1,2]. However, lowering of the mean HbA1c in DCCT (Diabetes and Control Trial) increased the rate of severe hypoglycemia threefold [1]. Despite technological progress such as self monitoring, use of insulin delivery devices (pens, pumps) the average glycemic control in type 1 diabetes is poor. In Sweden mean HbA1c in adults is about 7% (Mono-S method, corresponding to about 8% DCCT and 63 mmol/mmol, IFCC).

The blood glucose excursions in type 1 diabetes are a function of the input of glucose from food, mainly carbohydrates in the form of easily dissolved starch and sugars, and insulin from subcutaneous insulin stores. The estimation of the amount of carbohydrates in a meal has an error rate of 50% [3]. The insulin absorption may vary by up to 30% [4]. It is therefore virtually impossible to match carbohydrates and insulin which leads to unpredictable blood glucose levels after meals. By reducing the carbohydrates and insulin doses the size of the blood glucose fluctuations can be minimized. The risk of hypoglycemia is therefore minimized as well [5,6]. Around-the-clock euglycemia was seen with 40 g carbohydrates in a group of people with type 1 diabetes [7].

**Figure 1:**
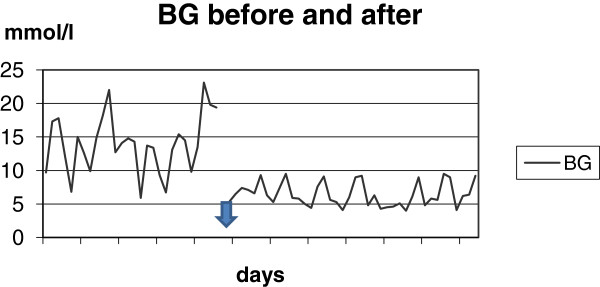
**An example of blood glucose values in one person before and after a reduction of carbohydrates and insulin.** The arrow marks the change. There are 25 measurement over 6 days before the change and 39 measurements over 7 days after. Mean BG in the left side is 14.0 mmol/l (range: 5.9 to 23.1 mmol/l). Mean BG in the right side is 6.4 mmol/l (range: 4.0 to 9.5 mmol/l). It is evident that lowering of the mean blood glucose in the left side of the figure is impossible without hypoglycemia. Flattening out of the small spikes in the right side can be done safely.

The immediate resulting stable, near-normal blood glucose levels allow individuals to predict after-meal glucose levels with great accuracy (see Figure 1). This regimen is little used, but can be an added important approach for some patients. Also in type 2 diabetes is reduction of dietary carbohydrates a useful regimen with lasting effect on all aspect of the metabolic syndrome [8].

For individuals with type 1 diabetes one year audit/evaluation of group education in this regimen has shown that the short-time lowering of mean HbA1c by 1 percentage unit and the reduction in mean rate of symptomatic hypoglycemia by 82% was maintained [9].

All individuals attending the educational course here have sought it themselves after having had written information of the regimen. It is practical guidance based on the central medicine sciences of physiology, biochemistry and pharmacology. It is aimed at normalizing the blood glucose [5,6]. This approach in almost all individuals gives the sought effect immediately. But since participants in diet studies generally show very poor adherence to whatever diet they are put on, we have again done an audit. The purpose is to map adherence among the individuals with type 1 diabetes, who attended the educational course at least 4 years earlier.

## Methods

### Attendees

The attendees were all outpatients. All individuals with HbA1c > = 6.1% (DDCT: 7.1%) attending the educational course from the start of 2004 to 2006 and adhering to it for at least the first 4 weeks are reported here. All patients, dissatisfied with their own glucose control and looking for ways to improve it, have received complete information about carbohydrates’ effect on their blood glucose and the effect of a reduction. It was then up to themselves to decide whether they wished to make any changes.

Individuals attended the course in groups of 5-7 people. The course consisted of one whole day followed by 4 sessions lasting 2-3 hours once a week over 4 weeks. The regimen is a carbohydrate restricted diet (carbohydrates 75 g/day or less) in combination with correspondingly adapted insulin doses. The method is described elsewhere [9]. An additional file shows the methods in more detail [see additional file 1].

### Adherence

Adherence was estimated from both HbA1c changes and an individual’s self reported adherence. The blood glucose changes the first days show the effect of the regimen; HbA1c after 3 months shows the effect of short-time adherence. From then on we have assessed adherence by comparing individual HbA1c values with the start and 3-months values. HbA1c continuously at 3 months level or lower suggests good adherence, and vice versa. Information about adherence to the diet was obtained from each individual’s own report in the clinical chart.

### HbA1c

Some people have a seasonal variation of HbA1c. The difference in HbA1c over a year appears to be related to the difference in temperature [10]. For each person we have therefore primarily used HbA1c taken the same months each year. If a value was missing the mean of the two adjacent HbA1cs are used.

HbA1c measurements were done in the same laboratory using the Mono-S method used in Sweden. This method gives values approximately 1 percentage unit lower than DCCT units. HbA1c (Mono-S) of 6.0% and 8.0% corresponds to HbA1c (DCCT) of approximately 7.0% and 9.0% respectively. This corresponds to 52 mmol/mol and 84 mmol/mol in IFCC units which is used in some places of Europe (IFCC = International Federation of Clinical Chemistry and Laboratory Medicine). Normal values for people below 50 years are: (Mono-S) < 5.5%, (DCCT) <6.5% and (IFCC) <46 mmol/mol.

Normal/near normal (<6%) HbA1c values cannot be expected to change much and are therefore not suitable to evaluate adherence in this report. Mono-S (%) to IFCC (mmol/mol) =10.45*[HbA1c]-10.67.

### Statistics

Means are given with standard deviation. Two-tailed *t*-test for dependent samples is used.

## Results

Altogether 48 persons with HbA1c > 6.1% (DCCT: 7.1%) had attended the course, 31 women and 17 men. This is 16% of all the patients connected to the present diabetes unit. Three persons came the first day but cancelled the next visit. All the individuals were known to us. Most had attended diabetes education before in the form of diabetes schools, diabetes camps etc. Fourteen used insulin pumps. There was no relation between the use of the pumps and HbA1c. Mean age and diabetes duration was 52 ±11.5 years and 24.0 ± 12 years respectively. Seven individuals with gatro-paresis used domperidone (motilium) in order to improve gastric motility.

### Weight, lipids and insulin

Table 1 shows mean weight and Body Mass Index (kg/m^2^) (BMI) and lipids. In the group of adherent people following changes were seen after 4 years: Mean Tot-Chol. from 5.3 ± 0.9 to 5.6 ± 0.8 mmol/l (p = 0.04). The increase was caused by an HDL increase from 1.5 ± 0.5 to 1.8 ± 0.4 mmol/l (p = 0.04). TAG was almost unchanged. Ratio Chol/HDL was 3.6 and 3.2 (p = 0.035); ratio TAG/HDL was 0.5 and 0.4 (P = 0.04) at start and after 4 years respectively.

**Table 1:** Mean weight and lipids in 48 patients with type 1 diabetes before and after 4 years of carbohydrate reduction

	**Start**	**3 months**	**P(1)**	**4 years**	**P(2)**
Weight(kg)	**77.6** ± 15	**74.9** ± 13.9	<0.001	**76.7** ± 14.6	0.18
BMI (Kg/m^2^)	**25.9** ± 3.5	**25.0** ± 3.4	<0.001	**25.7** ± 3.8	0.22
Tot-cholesterol (mmol/l)	**5.4** ± 1	**5.6** ± 1.0	0.2	**5.6** ± 0.9	0.44
HDL-cholesterol (mmol/l)	**1.5** ± 0.4	**1.6** ± 0.5	0.03	**1.7** ± 0.4	<0.001
Triacylglycerol(TAG)(mmol/l)	**0.9** ± 0.8	**0.8** ± 0.5	0.04	**0.9** ± 0.4	0.73
Ratio Chol/HDL	**3.9** ± 1.2	**3.5** ± 1.4	0.11	**3.5** ± 1.0	<0.001
Ratio TAG/HDL	**0.8** ± 1.2	**0.6** ± 0.9	<0.001	**0.6** ± 0.5	0.08

P(1) the p-value is for the difference between start and 3 months. P2) the p value is for the difference between start and 4 years. TAG: triacylglycerols; HDL: HDL-cholesterol; Chol: cholesterol.

The carbohydrates amount was reduced to 75 g/day or lower according to the patient’s own preferences. The insulin doses reduced according to the quantity of carbohydrates.

Meal insulin was reported for 36 patients. Mean daily dosage changed as follows: at start 23 ±9 IU; 1 year 13 ± 6 IU. After 4 years the doses were only sporadically reported. We have therefore not reported a mean. Mean long-acting insulin: there was a modest change from 19.6 ± 5 IU to 18.6 ± 6 IU the first year.

### HbA1c

Forty eight persons with a HbA1c >6.1 attended the course and could, based on HbA1c response and their own information in the chart be divided in non-adherent and adherent individuals. All achieved the expected mean HbA1c reduction after 3 months. After 2 years and onwards however, 25 persons (52%) had reverted with respect to both mean HbA1c and diet. There are 4 designations in table and figure. Their meanings are: A = all patients; B = non-adherent; C = partly adherent with intra-person variations over time; D = adherent.

**Figure 2:**
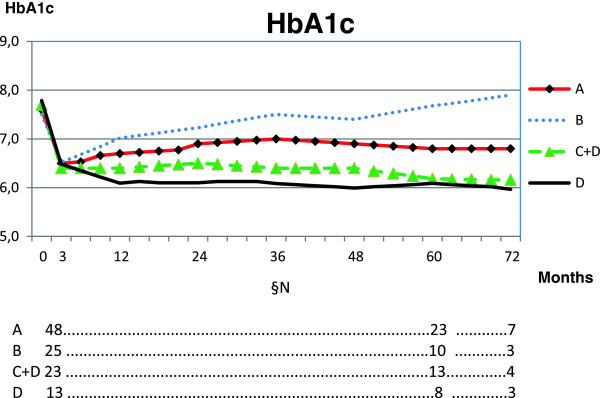
**Mean HbA1c over 4 years after reduction of carbohydrates in all and in subgroups with different degrees of adherence.** The amount of carbohydrates was 75 g/day or less according to the person’s own preferences. Mainly, starchy food was reduced. The insulin doses were adapted accordingly. §N number of patients in groups. A = all patients (100%). B = non adherent (52%). C = partly adherent with intra-person variations over time. D = excellent adherence (27%). C + D (47%).

The HbA1c changes in the groups are seen in Table 2 and Figure 2.

**Table 2:** Mean HbA1c before and 4 years after carbohydrate reduction in type 1 diabetes in groups with different degrees of adherence

**§Group**	**n**	**%**	**start**	**3 mo.**	**4 years**	*Change	**%**	**!p**
All	48	100	**7.6** ± 1.0	**6.3** ± 0.7	**6.9** ± 1.0	-0.7 ± 1.1	-8.4 ± 13	<0.001
B	25	52	**7.5** ± 1.1	**6.5** ± 0.8	**7.4** ± 0.9	-0.1 ± 0.3	-0.7 ± 11	0.7
C + D	23	48	**7.7** ± 1.0	**6.4** ± 0.9	**6.4** ± 0.8	-1.3 ± 0.9	-16 ± 10	<0.001
C	10	21	**7.6** ± 1.0	**6.3** ± 0.8	**6.9** ± 0.8	-0.7 ± 0.4	-8.8 ± 4	<0.001
D	13	27	**7.8** ± 1.0	**6.5** ± 0.8	**6.0** ± 0.6	-1.8 ± 0.9	-22.4 ± 8	<0.001

§ A: all patients. B: non-adherent patients. C: partly adherent with intra-person variations over time. D: with initial high HbA1c and excellent adherence. * The HbA1c change between start and 4 years in HbA1c units. ! P-values are for differences between start and 4 years.

Carbohydrates (mostly starch) were reduced to 75 g/day or lower. Insulin doses adapted to the quantity of carbohydrates.

### Cardiovascular disease

One person underwent percutanous coronary intervention in the 4 years.

## Discussion

After 2 years about half of the individuals had ceased adhering. The other half (group C + D) achieved a stable lowering of HbA1c. This is clinically significant. The tissue destructions from hyperglycemia accumulate steeply in the type 1 diabetes population [1]. The HbAc1 reduction therefore, even in this little group, already after a few years corresponds to a number of people avoiding complications [1].

The calculated number of persons who have avoided complications over 4 years among the 23 persons are: Retinopathy progression 2-3 persons (8-11%); laser treatment 1 person (4%); neuropathy 5-6 persons (22-26%); severe retinopathy 1 (4%). About 1 person (4%) per 5 years can be expected to avoid macular edema, and 1 person to avoid a cardiovascular event [1,2].

The ratio Chol/HDL is a marker of a specific risk for myocardial infarction [11]. The changes here correspond to a 20% reduction in this risk. The difference in HbA1c reduces the risk of cardiovascular disease by about 40% [1,2]. Substitution of fat for carbohydrates is generally more beneficial for risk of cardiovascular disease in both normal- and overweight persons than the widely recommended low-fat diet [12].

There is no reason to assume that the patients here are different from the rest of Sweden. The number of adults with type 1 diabetes in Sweden is about 40,000 and the number cared for by the present diabetes unit is about 300.

The 23 persons in group C + D constitute about 7.6% of all persons with type 1 diabetes cared for by the present unit. The figures in group C + D correspond to about 3,100 adults with type 1 diabetes in Sweden who might be expected to adapt to the present approach and avoid a substantial number of complications.

A small group D, 27% of the subjects, without exception adapted excellently and achieved a constant mean HbA1c lowering by 1.8 percentage point. This group constitutes about 4.3% of all individuals with type 1 diabetes connected to the present unit, and corresponds to about 1,700 of all adults with type 1 diabetes in Sweden.

Only a limited number of patients, about 16-18%, in contact with the present unit have been interested in such a change of diet as described.

There is no evidence for the use of the widely recommended high-carbohydrate, low-fat diet in type 1 diabetes.

There is no evidence that animal fat in the food should cause cardiovascular disease [13-15]. There is no evidence that protein should cause kidney disease [16]; on the contrary, hyperglycemia gave a 3.5 times higher incidence of albuminuria in DCCT, not protein [1]. There is, however, strong evidence for the aggressive development of damages in all organs in poorly regulated type 1 diabetes [1].

The physician and the individual must therefore together explore the tools and methods that give best result, for instance type of insulin, insulin pens, insulin pump etc. and diet. The restricted carbohydrate dietary approach is directly aimed at lowering of HbA1c, not at avoiding fat and protein. The model described here may be an option for 10-20% of the patients with type 1 diabetes.

## Conclusion

An educational program involving a low-carbohydrate diet and correspondingly reduced insulin doses for informed individuals with type 1 diabetes gives acceptable adherence after 4 years. One in two people attending the education achieves a long-term significant HbA1c reduction.

## Competing interests

The authors declare that they have no competing interests.

## Authors’ contribution

JVN wrote the manuscript and analysed the data. All authors collected data and gave final approval to the manuscript.

## Supplementary Material

Additional file 1 method details. Click here for file
