# Supplementary material for: Low carbohydrate diet in type 1 diabetes, long-term improvement and adherence: A clinical audit
Source: Diabetol Metab Syndr. 2012 May 31;4:23. doi: 10.1186/1758-5996-4-23 (PMC3583262; doi:10.1186/1758-5996-4-23)
Supplement: Additional file 1 — method details. [file 1758-5996-4-23-S1.pdf]

Additional file.

Intervention

The actual course consists of about 6-7 hours on the first day. They are split between 4 hours group-session and 2-3 hours individual advice. Two days later follows 2-3 hours group- and individual advice. The following 3 weeks the group meets once a week 2-3 hours; the time is divided between group- and individual advice. The blood glucose is normally stable the first afternoon. The follow-up meetings are used to individually tailor the doses. The attendees had already received written material necessary to understand the method in advance of the course. The attendees come fasting in the morning of the first day. They then eat breakfast and later lunch. The titration of insulin doses starts immediately in the morning. As a rule-of-thumb we assume that 1 unit subcutaneous rapid-acting insulin corresponds to 8 g glucose eaten. This is in most cases correct but in some people needs to be modified according to the blood glucose response. For calculation of meal doses as well as doses needed to correct too high values please see reference [6].

In a flow sheet the participants entered their own notes of blood glucose, time of each meal, amount of carbohydrates, insulin timing and doses, hypo- or hyperglycemias, the number of glucose table needed to correct a below-target blood glucose, the number of insulin units needed to correct an above-target pre-meal and bedtime blood glucose.

Regimen: A carbohydrate restricted diet (75 g/day or less) that excludes starchy food such as potatoes, rice, pasta, bread, cereal etc. that are quickly converted to glucose in the gut. The diet includes hard bread and vegetables. The diet here is therefore a glucose restricted diet. The patients are instructed to eat 3 meals per day, and abstain from eating between meals. To assist them the patients were given samples of recipes and menus showing the carbohydrate, protein and fat content in grams. The recipes are flexible; the carbohydrate content can be

modified according to the individual's own preferences. The initial distribution of carbohydrates, protein and fat in the samples was about15-20: 30:50-55 E% respectively. However, these samples are only for learning purposes. Each individual has personal preferences regarding protein and fat quantities and are advised to modify these dietaty components to suit themselves.

Insulin

Patients without insulin pump were switched to Aspart in a pen device (NovoRapid Pen for children) that enables delivery of half-units.

The insulin treatment consists of two arms: basal (long-acting insulin or continuous subcutaneous infusion of rapid-acting insulin from pump) and rapid-acting meal insulin. A correct basal dose allows the patient to postpone meals or not eat at all for 24 hours or more without symptomatic hypo- or hyperglycemia. Fast-acting insulin is taken to meals in small doses calculated from the amount of carbohydrates and protein.

Gastroparesis

Patients with gastroparesis and verified autonomous neuropathy were prescribed Domperidone (motilium) together with appropriate dietary adjustments.
